# 影像计划和实时引导肺癌穿刺活检在生物标志物检测中的进展

**DOI:** 10.3779/j.issn.1009-3419.2023.106.15

**Published:** 2023-08-20

**Authors:** Gengshen BAI, Bingyin ZHU, Jun MA, Yongchun LI, Gang HUANG, Yaqiong MA

**Affiliations:** ^1^730900 白银，白银市第二人民医院介入科; ^1^Department of Interventional, Baiyin Second People's Hospital, Baiyin 730900, China; ^2^730030 兰州，甘肃省人民医院放射科; ^2^Department of Radiology, Gansu Provincial Hospital, Lanzhou 730030, China

**Keywords:** 肺肿瘤, 活检, 生物标志物检测, 影像, Lung neoplasms, Biopsy, Biomarker detection, Image

## Abstract

随着肺癌个体化治疗的进展，临床对肺癌穿刺活检样本的要求越来越高。一个理想的肺癌活检样本不仅可以实现组织病理学诊断，还能够用于生物标志物检测。理想的活检样本需符合至少60 mm^2^的肿瘤组织和其内的瘤细胞占比在20%以上。要想获取理想的肺癌穿刺活检样本，就需要先进的影像技术予以帮助。本文就基于生物标志物检测对活检样本的要求及影像在肺癌穿刺活检术前计划穿刺路径及术中实时引导穿刺活检的现状和研究进展予以综述。

## Abstract (en)

With the progress of targeted therapy and immunotherapy for lung cancer, the clinical demand for lung biopsy is increasing. An ideal biopsy specimen can be used not only for histopathological diagnosis, but also for biomarker detection. The ideal biopsy specimen should meet two requirements, including more than 60 mm^2^ of tumor tissue and containing more than 20% of tumor cells. In order to obtain ideal lung cancer biopsy specimens, advanced imaging techniques are needed to help. In this article, we reviewed the requirements for biopsy specimens based on biomarker detection, as well as the current status and research progress of using imaging techniques for preoperative planning and intraoperative real time guidance of lung cancer biopsy.

肺癌是全世界发病率第二和死亡率第一的恶性肿瘤，大约2/3的肺癌被发现时已经处于中晚期，但随着肺癌靶向治疗、免疫治疗等精准治疗的飞速发展，肺癌患者的生存率得到了大幅度提高^[1]^。精准治疗的前提是精准诊断，而精准诊断需要活检样本。近些年，虽然液体活检样本得到了广泛的应用，但活检样本的金标准仍是组织样本^[2]^。一个理想的活检组织样本需满足两个要求，即组织样本大小必须大于60 mm^2^且样本内瘤细胞含量≥20%，才能行肺癌病理组织亚型分类、基因检测、免疫检查点检测以及耐药基因检测等^[3,4]^。肺癌瘤体内的异质性和肺癌周围的阻塞性病变、炎性病变及纤维化病变对获取一个理想的肺癌穿刺活检样本造成了诸多障碍^[5⇓-7]^。因此，近年来国内外有大量的学者在关注肺癌穿刺活检组织样本是否能满足生物标志物检测^[8]^及采用何种措施能够提高其充分性^[9⇓⇓-12]^。本文就基于生物标志物检测对肺癌活检样本的要求及影像在指导肺癌穿刺活检术中的现状和研究进展予以综述。

## 1 生物标志物检测对肺癌活检组织样本的要求

### 1.1 基因检测对活检组织样本的要求

用于基因检测的样本是否理想由两个因素决定：肿瘤组织的大小和肿瘤细胞含量^[13]^。选择的富肿瘤区的大小和其内活肿瘤细胞的数量决定了DNA产量，这是二代测序（next generation sequencing, NGS）的关键质量控制步骤。单个有核细胞的预期平均DNA产量约为6 pg，为了获得离子AmpliSeq癌症热点panel推荐的10 ng DNA，大约需要2000个细胞，相当于需要一个未染色的福尔马林固定的石蜡包埋（formalin-fixed paraffin embedded, FFPE）切片上至少含60 mm^2^的富肿瘤组织，才能产生足够的DNA进行NGS突变分析，如果选择的切片上富肿瘤组织小于10 mm^2^ ，即使选择多个（10-20）未染色的FFPE组织一起提取组织，也不能提供足够的DNA^[3]^。在所选肿瘤组织切片极小的肿瘤中，由于DNA数量不足而导致的NGS失败率通常比所选肿瘤组织切片较大的肿瘤中要高得多^[14]^。通过选择更大的区域来增加组织大小可能会增加DNA产量，但这种增加通常包括更多的非肿瘤成分，如基质细胞。当肿瘤占比低于NGS平台的分析灵敏度（约5%）时，假阴性的风险很高^[3]^。使用靶向酪氨酸激酶抑制剂治疗非小细胞肺癌（non-small cell lung cancer, NSCLC）患者的分子检测指南^[4]^中指出：在NGS分析中，通常需要20%或更多的肿瘤细胞含量。因此，为了实现基因分析，穿刺活检样本FFPE切片上既要包含至少60 mm^2^的肿瘤组织，其内的瘤细胞占比也要在20%以上。

### 1.2 免疫检查点检测对肺癌活检组织样本的要求

除了基因检测对肺癌穿刺活检样本有要求，免疫检查点检测对活检组织样本同样有要求。目前，临床常采用免疫组化（immunohistochemistry, IHC）检测程序性死亡配体1（programmed death ligand 1, PD-L1）的表达。Kitazono等^[15]^使用79对NSCLC小活检样本和手术切除的标本分别检测PD-L1的表达，结果表明样本之间的一致性良好。为了提高小活检样本检测PD-L1表达的可靠性，IHC检测实体肿瘤PD-L1指南提出了在22C3和28-8检测系统上检测，染色切片上至少要有100个活肿瘤细胞，在SP142检测系统上检测，至少需要50个活的肿瘤细胞和/或肿瘤相关基质^[16]^。基于组织样本应该包含至少100个肿瘤细胞这样的标准，Heymann等^[17]^指出IHC在检测PD-L1表达中，小活检样本与手术切除标本没有显著差异（P=0.99），Tsunoda等^[18]^也得出了相同的结果，当使用较小的样本评估PD-L1表达时，肿瘤比例评分的频率与过去使用较大样本的临床试验相当。

然而，Bigras等^[9]^发现，100个肿瘤细胞（相当于2 mm^2^）的阈值会导致高达35%的PD-L1分类错误，并提出采用5 mm^2^的阈值[代表一个芯针切割（core-needle biopsy, CNB）切割活检组织面积为10 mm×0.5 mm]取代当前的100个肿瘤细胞的阈值，可降低约25%的PD-L1分类错误。因此，活检组织越大，PD-L1错误分类就越低；活检组织较小时，会增加PD-L1的假阴性或错误分类。

## 2 穿刺技术因素对肺癌活检样本能否满足生物标志物检测的影响

### 2.1 细针抽吸（fine-needle aspiration, FNA）和CNB活检样本

与手术切除大标本相比，FNA和CNB样本不仅一开始就小，产生的DNA少，而且随着后续分子生物标志物测试切片的消耗，它们往往会变得更小。因此，当严格遵循制造商推荐的DNA输入量时，FNA和CNB样本的NGS成功率低于手术切除的大标本^[14]^。但是，大约2/3的肺癌被发现时已处于中晚期，只能通过FNA和CNB等微创手术获得组织样本。因此，采用将输入的DNA阈值降低，一部分高质量DNA但低DNA浓度的病例也可以在离子流平台上成功测序，FNA和CNB样本就能够得到与手术切除标本一致的NGS成功率^[14,19]^。但是，一项meta分析^[8]^报道，FNA和CNB样本能够行基因检测的充足率是89.7%，意味着仍然有10.3%的穿刺活检样本不足以进行基因检测。Schneider等^[11]^和Gill等^[20]^的研究结果显示，FNA样本进行基因分析的充分率仅为46%和78%，均低于CNB样本（67%和90%）。这可能是因为FNA获得的样本是细胞而不是组织，不能满足基因分析对组织大小的要求。然而，Coley等^[21]^在基因分析的评价中没有观察到FNA和CNB样本之间的这种差异，可能是由于所有穿刺活检中都应用了快速现场评估（rapid on-site evaluation, ROSE）。除了在手术过程中提供即时的评估外，ROSE还强调了组织的获取、分诊和辅助检测的分配。因此，如果ROSE可以用于每个病例，那么，放射科医生可以根据对病变特征和肺部状况的综合评估来选择FNA或CNB，如患者肺部存在肺气肿、肺大疱、肺间质纤维化等基础状况较差的情况，且肺内病灶为实性病灶，可推荐采用FNA，以减少气胸、肺内出血等并发症的发生；如肺内病变为亚实性病灶，切割一次亚实性病灶容易导致出血，而影响穿刺针与病变实性成分的具体位置观察，因此在患者肺基础状况较好的情况下，推荐采用CNB，尽量达到一次切割组织即可获取一条较粗大的组织样本，以弥补亚实性病灶切割组织内肿瘤细胞不足的情况。

### 2.2 穿刺活检针的大小和切割组织次数对活检样本能否满足生物标志物检测的影响

对于针径粗细和切割组织次数对基因分析的影响还没有明确的共识。Cheung等^[22]^的研究表明在18 G CNB获取的组织样本中提取DNA的浓度（平均为 47.13 ng/μL）高于20 G CNB（平均为35.92 ng/μL），但他们的研究也表明无论使用18 G活检针还是20 G活检针，所获得的组织样本均可以成功实现基因分析。然而，Elsakka等^[10]^发现，小口径针头（≥20 G）与肺癌中NGS测序失败的可能性独立相关。那么，对于切割组织次数增加与穿刺活检针内径增粗这两个因素而言，哪个因素对于核酸数量的增加影响更大呢?Jamshidi等^[23]^报道，使用18 G活检针穿刺一次肿瘤组织产生的核酸数量比使用20 G活检针多4.8-5.7倍。因此，如果能够安全地到达目标部位，那么18 G活检针将更适合于用于NGS基因分析。

## 3 肺癌瘤体内的异质性对生物标志物检测的影响

无论从大体病理还是从肿瘤微环境的角度来看，肺癌都是高度异质性的肿瘤^[24,25]^。在肿瘤内部，存在着由灌注、缺氧、细胞密度、增殖等生物因素引起的较大的空间变化。Hiraki等^[5]^和Yeow等^[6]^研究表明，随着肺癌肿瘤直径的增大，肿瘤内部坏死组织也增加，容易导致活检样本中肿瘤细胞不足，从而导致诊断失败。因此，当肺癌的肿瘤较大时，由于样本中肿瘤细胞含量较少，通过肺癌穿刺活检获得的组织标本可能不足以进行生物标志物检测。通过经皮肺癌穿刺活检获得的组织样本进行精准诊断的成功率见表1。Cooper等^[26]^和Foo等^[27]^表明生物学上的异质性导致了功能和代谢上的异质性。在这种情况下，有研究^[28]^认为，在细胞数量更多或代谢程度更高的区域可能存在相关的基因突变，而这些突变可能不存在于同一病变内的其他区域。在PD-L1 检测中，专家共识要求应避免选择含有坏死组织、挤压细胞的样本^[16]^。因此，穿刺活检时根据功能影像或代谢影像所指示的细胞密集区或高代谢区进行穿刺，结果会更加有效，患者的受益也应该更大。

**表1:** 经皮肺穿刺活检样本在肺癌精准诊断中的成功率

Purpose	Precision diagnosis	Primary disease	Guide methods	Type of needle	Needle size (G)	The availability of ROSE	Successful rate for biomarkers detection and other results	Reference
Effect of CNB and FNA specimens on gene analysis	*EGFR, ALK, KRAS*	Lung adenocarcinoma	CT	CNB FNA	18-22 G 22-25 G	NA NA	35/52 (67%) 55/120 (46%)	Schneider F, 2015^[11]^
	*EGFR, KRAS*	NSCLC	CT, CTF	CNB FNA	18 G or 20 G 22 G	NA NA	457/509 (90%) 53/68 (78%)	Gill RR, 2018^[20]^
	*EGFR, ALK, KRAS*	Lung adenocarcinoma	CT	CNB FNA	NA NA	Yes Yes	19/19 (100%) 16/17 (94%)	Coley SM, 2015^[21]^
	NGS	Lung cancer	CT, CTF, US, PET/CT	CNB FNA	18 G or 20 G 20 G or 22 G	Yes Yes	428/470 (91%) 5/9 (56%)	Elsakka A, 2022^[10]^
Effect of needle size on gene analysis	Concentration of DNA extract	NSCLC	CT	CNB CNB	18 G 20 G	NA NA	Average 47 ng/μL Average 36 ng/μL	Cheung YC, 2010^[22]^
	NGS	Lung cancer	CT, CTF, US, PET/CT	CNB, FNA CNB, FNA	18 G 20 G	Yes Yes	255/268 (95%) 178/211 (84%)	Elsakka A, 2022^[10]^
	Nucleic acid yields	Lung cancer	CT	CNB CNB	18 G 20 G	NA NA	(1840±405) ng (383±68) ng	Jamshidi N, 2017^[23]^
Effect of CNB specimens on PD-L1 detection	PD-L1	Lung cancer	CT	CNB	NA	NA	6 (92%)	Kitazono S, 2015^[15]^
	PD-L1	NSCLC	CT	CNB	NA	NA	39 (96%)	Heymann JJ, 2017^[17]^
	Tumor cell counts for TPS	Lung cancer	CT, US	CNB	20 G	NA	6593 (100-37,500)	Tsunoda A, 2019^[18]^
	PD-L1	NSCLC	NA	CNB	NA	NA	65%	Bigras G, 2018^[9]^

EGFR: epidermal growth factor receptor; ALK: anaplastic lymphoma kinase; KRAS: kirsten rats arcomaviral oncogene homolog; CT: computed tomography; CTF: CT fluoroscopy; US: ultrasound; PET/CT: positron emission tomography/CT; NSCLC: non-small cell lung cancer; TPS: tumor proportion score; NGS: next generation sequencing; CNB: core needle biopsy; FNA: fine needle aspiration; ROSE: rapid onsite evaluation; G: gauge; NA: not available.

## 4 不同的影像计划和实时引导方式对经皮肺癌穿刺活检结果的影响

对于异质性较大的肺癌或者伴阻塞性病变的肺癌而言，为了获取的活检组织样本内含足够的瘤细胞，一般采用两种方法：（1）穿刺活检术前通过先进的影像技术评估计划穿刺路径^[28⇓⇓⇓⇓-33]^；（2）穿刺术中通过先进的影像技术实时引导穿刺针精准进入肿瘤内的有效瘤组织中^[34⇓⇓⇓-38]^。目前，在穿刺活检术前计划穿刺路径时，常采用增强计算机断层扫描（computed tomography, CT）、正电子发射计算机断层扫描/CT（positron emission tomography/CT, PET/CT）、磁共振扩散加权成像（magnetic resonance-diffusion weighted imaging, MR-DWI）和能量CT成像评估肿瘤内的异质性及肿瘤与周围阻塞性病变的界线。穿刺活检术中常用的影像实时引导方式是平扫CT，术者多通过视觉匹配术中平扫CT图像与术前增强CT图像、PET/CT图像或者MR-DWI图像来进行肺癌穿刺活检以达到精准穿刺的目的^[28,29]^；在具备图像融合软件的前提时，术者更倾向于采用术中平扫CT图像与PET/CT图像或MR-DWI图像融合来指导肺癌精准穿刺活检^[30,33]^。对于复杂的肺癌病例，有较多学者研究了利用PET/CT、MR实时引导肺穿刺活检，虽然明显提高了活检样本的准确性和充分性，但在临床工作中，因价格昂贵、操作不便等原因并未得到广泛应用。近两年，在精准穿刺活检中，能量CT成像于术前计划穿刺路径已初露锋芒。不同的影像计划和实时引导方式对经胸肺癌穿刺活检结果的影响是不同的（表2）。

**表2:** 不同的影像计划和实时引导方式对经皮肺癌穿刺活检结果的影响

Findings	Design	Methods	Sample size	Type of needle	Results			Reference
					Sensitivity	Specificity	Accuracy	
CNB combined with PET/CT improves the rate of accurate diagnosis	A retrospective study	CT planning	26	CNB	73%	100%	NA	İntepe YS, 2016^[29]^
		PET/CT planning	20	CNB	91%	100%	NA	
CNB with prior PET/CT fusion imaging is particularly helpful in improving diagnostic yield and accurate rate of biopsy in lung masses	A prospective study	CT planning	69	CNB	NA	NA	75%	Lin Y, 2022^[30]^
		PET/CT planning	76	CNB	NA	NA	83%	
Anatomy- and metabolism-based FNA guidance using information provided by PET/CT may increase the probability of definitive diagnosis	A retrospective study	PET/CT planning	267	FNA	81%	90%	71%	Guralnik L, 2015^[31]^
MR-DWI and PET/CT showed that tumors are functional and metabolically heterogeneous	A prospective study	MR-DWI planning	14	CNB	58%	100%	64%	Zurstrassen CE, 2020^[28]^
		PET/CT planning	13	CNB	50%	50%	50%	
MR-DWI enables the collection of adequate material for specific diagnosis	A prospective study	MR-DWI planning	13	CNB	NA	NA	100%	Guimaraes MD, 2014^[32]^
DWI and the apparent diffusion coefficient, are promising for implementation in imaging-guided procedures	A prospective study	MR-DWI planning	8	CNB	NA	NA	100%	Guimaraes MD, 2014^[33]^
MRI-guided CNB is a safe and accurate diagnostic technique in the evaluation of small lung nodules	A prospective study	MR guidance	96	CNB	96%	100%	97%	Liu M, 2013^[36]^
MR-guided CNB is safe, feasible, and high accurate diagnostic technique for pathologic diagnosis of pulmonary nodules	A retrospective study	MR guidance	69	CNB	96%	100%	97%	Liu S, 2015^[37]^
MRI-guided percutaneous biopsy using a 1.0-T open MR scanner with respiratory gating is an accurate and safe diagnostic technique in evaluation of pulmonary lesions	A prospective study	MR guidance	65	CNB	96%	100%	97%	Liu M, 2017^[38]^
Spectral CT parameters can be used to identify regions with at least 20% tumor cell content in lung cancer for CNB	A prospective study	SDCT planning	82	CNB	71%	97%	82%	Ma Y, 2023^[12]^

MR-DWI: magnetic resonance-diffusion weighted imaging; SDCT: dual-layer spectral detector CT.

### 4.1 常规CT图像

常规CT图像包括平扫CT图像和增强CT图像。经皮肺癌穿刺活检术前均需增强CT图像对肿瘤内和肿瘤周围重要结构及微细结构进行评估^[39]^。增强CT图像可以很好地呈现出病灶周围的血管及瘤内不强化区，可以帮助术者避开重要结构及瘤内液化坏死区从而规划出一个安全且有效的活检路径。平扫CT引导是肺活检最常用的引导方式，优点包括简便、高空间分辨率、较好的对比度和准确的针尖定位^[40]^。术者通过视觉或图像融合软件匹配术中平扫CT图像和术前增强CT图像引导穿刺针进入肿瘤内的适当部位，避开明显的液化坏死区域。缺点是常规CT图像不易识别肿瘤内的凝固性坏死区、出血性坏死区、瘤周纤维化区、瘤周阻塞性病变等密度与瘤组织密度相似的非有效瘤组织，可能将穿刺针引导入肿瘤内或肿瘤周围无有效瘤组织的区域，随后出现穿刺结果呈假阴性或组织样本不足以行生物标志物检测的情况。

### 4.2 PET/CT成像

^18^F-FDG PET/CT成像可提供肺病变的代谢特征信息^[41]^，能够把恶性肿瘤内代谢旺盛的区域与纤维化或坏死组织分离开^[7]^。因此，在临床工作中，术者常根据术前PET/CT图像采用视觉匹配的方法计划穿刺路径，也有学者采用图像融合软件将穿刺活检术前PET/CT数据与术中平扫CT图像匹配融合，从而引导穿刺针尖直达肿瘤内细胞代谢旺盛的部位，增加获得明确诊断的机会，这种方法在进行CT引导CNB和FNA时均可以提供显著的增量效益^[31,42]^。但PET/CT的特异性受到各种非恶性病变中^18^F-FDG积累的阻碍^[43,44]^。另外，PET/CT价格昂贵，实时引导穿刺时还会导致活检操作医生受到核素辐射危害^[45]^。因此，PET/CT在穿刺活检术前计划穿刺路径中具有重要价值，但实时引导肺癌穿刺活检在临床实践中并未得到广泛应用。

### 4.3 MR成像

MR-DWI已经广泛用于评估肺病变^[46]^。DWI能够区分开高细胞密集度和低细胞密集度的区域^[47]^。相关文献^[48]^报道，肺癌瘤内组织的表观弥散系数值与PET/CT上的最大标准摄取值呈负相关。因此，MR在肺穿刺活检术前计划穿刺路径和术中引导时选择高细胞密集度区域取样具有极高的准确性、敏感度和特异度^[36]^。MR引导的另一个优点是无电离辐射。但是，MR-DWI不能细致地观察肺肿瘤周围的小血管、叶间裂和肺大疱等，这限制了肺穿刺活检术前穿刺路径的精准设计。另外，MR实时引导耗时，且抗磁穿刺针等耗材非常昂贵。因此，MR应用于术前计划穿刺路径或术中引导应考虑用于复杂的病例，包括肿瘤体积较大、瘤内异质性高的或合并阻塞性病变的肿瘤，在这些特殊情况下，相比于因为组织样本不足或假阴性样本需要二次活检所带给患者的风险，这种引导方法的时间和成本付出才是合理的。

### 4.4 能量CT成像

能量CT是指在两个或更多的能量下获得物质的衰减信息，不同的组织有不同的能量依赖性，因此，基于光子吸收的不同，可以对不同物质或组织进行区别和分类^[49]^。因为它能够提供传统CT不能提供的组织特征性信息，所以，在临床实践中越来越表现出优势和特色。特色一：低能级单能量图像可以更好地显示肿瘤与肿瘤周围组织的界线^[50]^，可以显示肺癌与阻塞性肺不张或阻塞性肺炎之间的界线^[51]^，由此推断，低能级单能量图像可指导伴阻塞性病变的肺癌的精准穿刺；Sekiguchi等^[52]^学者提出40 keV的虚拟单能图像可显示出肺门血管和肺门淋巴结之间的最大对比差异，因此，当用于计划肺穿刺活检路径时，有助于穿刺过程中避开肿瘤内及瘤周血管，避免肺内或胸腔内出血。特色二：能量CT碘密度图可用于评估肿瘤内的异质性^[53,54]^，因此，当用于计划肺穿刺活检路径时，可引导穿刺针避开肿瘤内的坏死区域，穿刺入肿瘤细胞密集或代谢旺盛的区域，使穿刺活检样本内有效肿瘤细胞比例更高。Ma等^[12]^研究发现双层探测器光谱CT（dual-layer spectral detector CT, SDCT）参数碘密度值能够识别出肺癌肿瘤内肿瘤细胞比例在20%以上的区域，从而帮助精准计划CT引导下肺穿刺活检路径。特色三：有效原子序数图可清晰显示出肿瘤组织与坏死组织、肿瘤周围炎性组织的界线，Curti等^[55]^提出，在个体化治疗时代，SDCT参数之有效原子序数图像可以用来指导经皮肺穿刺活检，从而提供更有效的诊断样本和生物标志物信息。图1的能量CT参数图碘密度图及有效原子序数图可清晰显示肺癌瘤内的异质性。

**图1:**
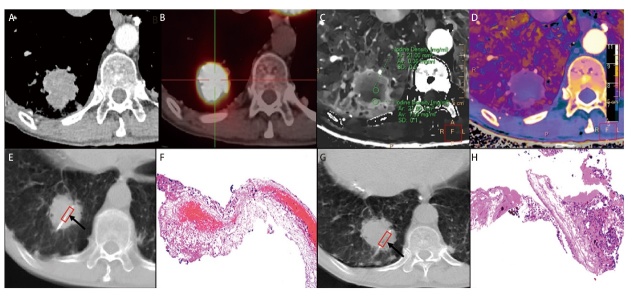
常规增强CT图像、PET/CT图像、能量CT参数图像指导肺癌穿刺活检。A-D：分别为常规动脉期CT图像、PET/CT图像、动脉期碘密度图像、动脉期CT_40 keV_/有效原子序数图融合图像。右肺下叶肿瘤常规增强CT上呈均匀密度，PET/CT上呈均匀高代谢。碘密度图显示肿瘤边缘碘密度为1.09 mg/mL，肿瘤中心碘密度为0.36 mg/mL。在CT_40 keV_图与有效原子序数图融合图像上，肿瘤内伪彩明显不均匀，肿瘤边缘颜色较肿瘤中心颜色亮。E：显示穿刺针槽（箭所指红框内）位于肿瘤中心。F：与E相对应的活检标本镜下放大图像（HE染色，×100），显示出血性坏死多且肿瘤细胞少，不足以明确诊断。G：显示穿刺针槽（箭所指红框内）位于肿瘤边缘。H：与G对应的活检标本镜下放大图像（HE染色，×100），显示有足够的肿瘤细胞可供明确诊断。由此可见，比起常规增强CT及PET/CT图像，能量CT碘密度图、CT_40 keV_图与有效原子序数图融合图像能够提供肺癌瘤内更清晰的细节，可精确指导肺癌穿刺活检。注：CT_40 keV_图为虚拟单能级40 keV图。

### 4.5 影像组学

不管是增强CT成像、PET/CT代谢成像、MR-DWI还是能量CT成像，都是肉眼观察图像和测量感兴趣区的参数值来选择靶目标区，它们所提供的信息都是有限的。而影像组学是指从影像图像中高通量地提取大量高维的定量影像特征，以创建可挖掘的数据库^[56]^。Gillies等^[57]^在一篇题为“影像组学：图像不仅仅是图片，它们是数据”的综述中指出影像组学可以帮助决定在肿瘤的哪个区域进行活检或切除。那么，定量分析肿瘤内不同区域的影像组学特征就可以准确地为活检提供信息，也就是说，影像组学特征可以预先识别出复杂肿瘤内最有可能包含重要的诊断、预后或预测信息的区域。这就显示了影像组学的潜力，可以为穿刺活检医生提供更好的信息以决定在肿瘤内的哪个区域进行活检。

## 5 总结与展望

精准治疗肺癌的前提是精准诊断，精准诊断需要有效的穿刺活检组织样本，为了获得组织大小和瘤细胞含量能满足生物标志物检测的组织样本，影像计划和引导肺癌穿刺活检是一个重要手段。增强CT、PET/CT和MR-DWI在异质性大的肺癌穿刺活检术前路径计划和术中引导中均具有显著价值。能量CT在对肿瘤内的功能与代谢评估方面，具有和PET/CT及MR相媲美的优势，并具有能够良好显示肺癌周围微细结构的优势，可能会成为肺癌穿刺活检术前计划和术中引导的首选方式。影像组学发展迅速，利用能量CT及影像组学构建肺癌精准穿刺活检模型可能会成为未来肺癌精准穿刺活检的重要方法。

**Competing interests**

The authors declare that they have no competing interests.
